# Morphological and Chemical Analysis of Low-Density Polyethylene Crystallized on Carbon and Clay Nanofillers

**DOI:** 10.3390/polym13101558

**Published:** 2021-05-13

**Authors:** Dilip Depan, William Chirdon, Ahmed Khattab

**Affiliations:** 1 Institute of Materials Research and Innovation, Department of Chemical Engineering, University of Louisiana at Lafayette, P.O. Box 43675, Lafayette, LA 70504-4130, USA; william.chirdon@louisiana.edu; 2 College of Engineering, University of Louisiana at Lafayette, P.O. Box 43675, Lafayette, LA 70504-4130, USA; khattab@louisiana.edu

**Keywords:** polyethylene, interfacial crystallization, carbon nanotubes, graphene, montmorillonite

## Abstract

Interest in carbon and clay-based nanofillers has grown in recent years. The crystallization behavior of low-density polyethylene (LDPE) was studied using a variety of notable nanofillers used in engineering applications and prepared using a solution crystallization method. Carbon nanotubes (CNTs), graphene oxide nano-platelets, clay (montmorillonite), and modified clay (surface-modified with trimethyl stearyl ammonium) were used to induce heterogeneous crystallization of LDPE. The crystallized LDPE samples, imaged using scanning and transmission electron microscopy, revealed different microstructures for each nanohybrid system, indicating these various nanofillers induce LDPE lamellae ordering. The underlying interactions between polymer and nanofiller were investigated using FTIR spectroscopy. X-ray diffraction (XRD) was used to determine crystallinity. This work examines how the differences in morphology and chemical structure of the nanofillers induce changes in the nucleation and growth of polymer crystals. These results will provide guidance on functional design of nano-devices with controlled properties.

## 1. Introduction

The role of nanofillers in modulating the physical properties of polymer nanocomposites has attracted tremendous interest from both industry and academia [1]. Since, the processing cycle of polymers is critically dependent on the rate of crystallization, a fundamental understanding of interactions between polymer and filler becomes crucial. However, the conditions for nucleation on the surface of fillers are not very clear, owing to the complex interactions between filler and polymer. Nonetheless, it is essential to understand the crystallization mechanism to design materials with tunable properties. Recently, considerable effort has been dedicated to controlling the crystallization via interfacial interactions between the polymer matrix and nanoparticles as their interactions are critical to improving the resultant mechanical properties of the nanocomposites [2].

Carbon nanotubes (CNTs) and graphene oxide (GO) have been considered to be effective reinforcement materials due to their exceptionally high specific surface area and desirable mechanical and electrical properties [3,4]. CNTs and GOs serve as heterogeneous nucleating agents for the crystallization of various polymers [5]. The presence of nanofillers is expected to influence the semicrystalline morphology and conformational changes, owing to different polymer chain orientation and crystal polymorphism [6,7,8]. Through control of the microstructure, carbon, and clay-based nanofillers have been found to improve the physical properties of polymers. Moreover, controlling the microstructure, carbon, and clay-based nanofillers have been found to improve the physical properties of polymers. Cellulose nanofibers, when embedded with highly ordered clay nanoplatelets, exhibited superior fire retardant properties [9]. Further, the effect of self-assembled MWCNTs was found to accelerate the electrical properties of shape-memory polymers [10].

Other materials under investigation for nucleating polymer crystallization include aluminosilicate clays such as montmorillonite. These nanoclays are layered silicates and are believed to be highly effective nucleation sites when dispersed properly, which would influence the crystallization rate of polymers [11,12]. Regardless of the type of nanofiller and/or nucleating agent added, the polymers experience a change in crystallization kinetics and crystal morphology as a result. The morphology of the nucleating agent can also impose the conformational and structural changes when the polymer crystallizes.

Studying the effect of different nanoparticles and their effect on the crystallization of PE is an interesting topic to investigate, which is evidenced by the large number of studies on the topic [13]. Most of these studies focus on the effect of nanofillers on the physical properties of the composite materials. However, results featuring direct comparisons of different nucleating agents with varied structures and their subsequent effect on crystallization of LDPE are relatively scarce. When crystallizing in the presence of a nucleating agent, Bai et al. have shown that the nucleating agent not only improves the crystallization kinetics, but also affects the crystalline morphology [14]. We recently reported the effect of various crystallization parameters such as temperature, time, and polymer concentration on nanohybrid shish-kebab formation on CNTs [15].

However, the interactions during interfacial crystallization and its effect on the polymer conformational changes are not well understood. This understanding is essential to fully exploiting the potential of carbon- and clay-based nanohybrids, because these interactions control nucleation and growth and a properly ordered interfacial microstructure significantly improves the interfacial adhesion and load transfer [16,17]. Different nucleating particle types can produce different crystal morphologies resulting in varied properties among the resultant composites. The objective of this work is firstly to determine the effects of various nucleating agents (CNTs, GO, clay, and modified clay) on the crystallization of LDPE. The surface morphology of LDPE crystallized on the various nucleating agents as well as the chemical interactions within the composites were compared in order to gather new insight into the origin of the nucleating effects and to quantitatively determine their impact on crystallization.

## 2. Materials and Methods

### 2.1. Materials

Low-density polyethylene (LDPE, EM460, with melt flow index of 27 g per 10 min; density 0.918 g·cm^−3^) was provided by Westlake Polymers Corporation (Houston, TX, USA, October 2018). Single-walled carbon nanotubes (SWCNTs) of outer diameter 2 nm and graphene oxide (GO) were obtained from Cheap Tubes Inc, Cambridge, MA, USA. GO had lateral dimension of 300–800 nm, with a thickness of 0.7–1.2 nm, and 99% purity, as per the supplier (Sigma-Aldrich, St. Louis, MO, USA). Natural clay (Montmorillonite SiO_2_/Al_2_O_3_), nanoclays (surface modified with 25–30 wt.% trimethyl stearyl ammonium), and decahydronaphthalene were obtained from Sigma-Aldrich, St. Louis, MO, USA. The BET specific surface area (m^2^·g^−1^) of CNTs, graphene oxide, clay, and modified clay were 407, 350, 250, and 750, respectively.

### 2.2. Methodology

The nucleating agents (CNTs, GO, natural clay, and modified clay) were first sonicated in decahydronaphthalene at a concentration of ~1 mg·mL^−1^ in a bath sonicator (Fisher Scientific FS-30 bath sonicator with 4-kHz frequency and 150 W power, Fisher Scientific, Pittsburgh, PA, USA) for 1 h at room temperature. Solution crystallization was carried out using the following steps: (1) LDPE was dissolved in decahydronaphthalene at 120 °C in a round bottom flask. (2) After complete dissolution of LDPE (~1 h), the dispersed nucleating agent (polymer to nanofiller ratio = 5:1) was then transferred drop-wise into the dissolved polymer. (3) The LDPE- nucleating agent mixture was then agitated for 5 min, followed by crystallization at 80 °C for another 30 min in an oil bath. (4) The flask was then removed from the oil bath and allowed to rest at room temperature for an additional 30 min. (5) The solution was then poured into a Petri dish and the solvent was allowed to evaporate under a fume hood until the sample was completely dry.

### 2.3. Characterization Techniques

Scanning electron microscopy (SEM) was used to study the surface morphology of the composites. Samples were sputter coated with gold (~12 nm deposition) prior to analysis using a JEOL J-800 field emission SEM (Peabody, MA, USA) at an operating voltage of 10 kV. The chemical structures of dried specimens were studied using an Agilent Cary 630 FTIR (Santa Clara, CA, USA) under ATR mode using a ZnSe crystal, with a 45 angle of incidence. 64 scans were acquired with a spectral resolution of 2 cm^−1^. Further imaging was completed using transmission electron microscopy (TEM, Krefeld, Germany) for a more detailed structural analysis of the samples. The samples were diluted in decahydronaphthalene and placed onto 400 mesh copper/carbon films (CF400-CU, Hatfield, PA, USA) from Electron Microscopy Sciences and analyzed using a Hitachi 7600 TEM (Krefeld, Germany) at 80 kV. X-ray diffraction (XRD) was used to determine the d-spacing and the percent crystallinity of each of the samples. The measurements were carried out in a MiniFlex 600 from Rigaku Americas Corporation (Woodlands, TX, USA). The full width half maximum (FWHM) values were also determined as well as the intensities of the peaks. The samples were measured over a 2θ range of 10–40°. The WXRD patterns were recorded by a Cu Kα radiation diffractometer set at λ = 1.542 Å, at 40 kV and 30 mA, with a step size of 0.02°, and a step time of 2.0 s.

## 3. Results and Discussion

### 3.1. Morphology of Crystallized Polymer on Various Nanofillers

#### 3.1.1. Polymer Crystal Structure on Carbon-Based Nanofillers

**Figure 1:**
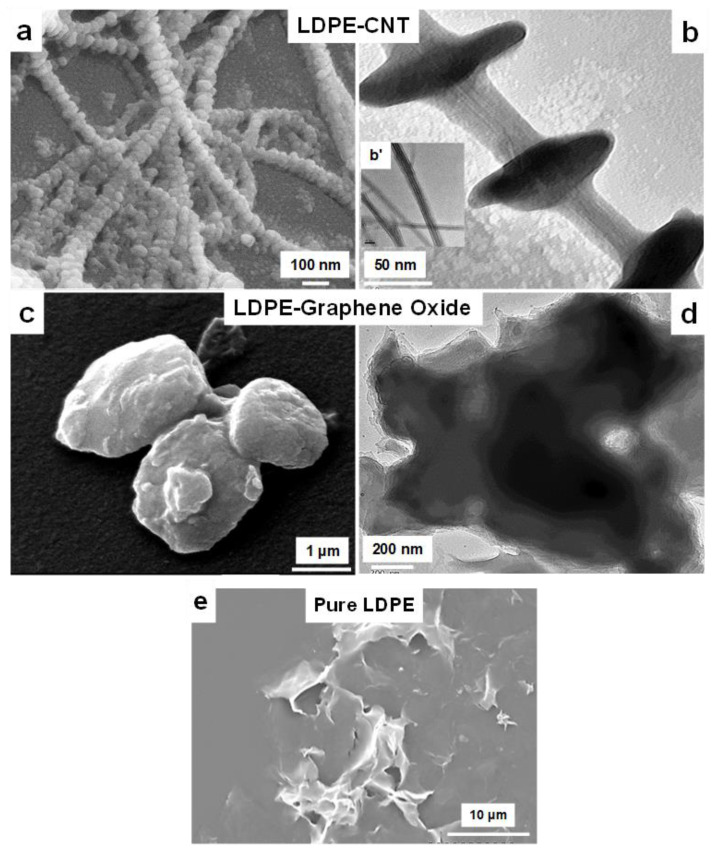
SEM (**a**) and TEM (**b**) micrographs of LDPE-CNTs, while (**c**,**d**) are corresponding micrographs of LDPE-GO. INSET images are AFM data of pure GO. Pure LDPE crystallized under similar conditions is given as (**e**). The magnifications in the micrographs are as: a, b and b’ (INSET): 90 k; c: 20 k; d: 60 k; e: 3 k. The SEM chamber pressure varied from 1.4 × 10^−6^ mbar–4 × 10^−6^ mbar.

**Figure 2:**
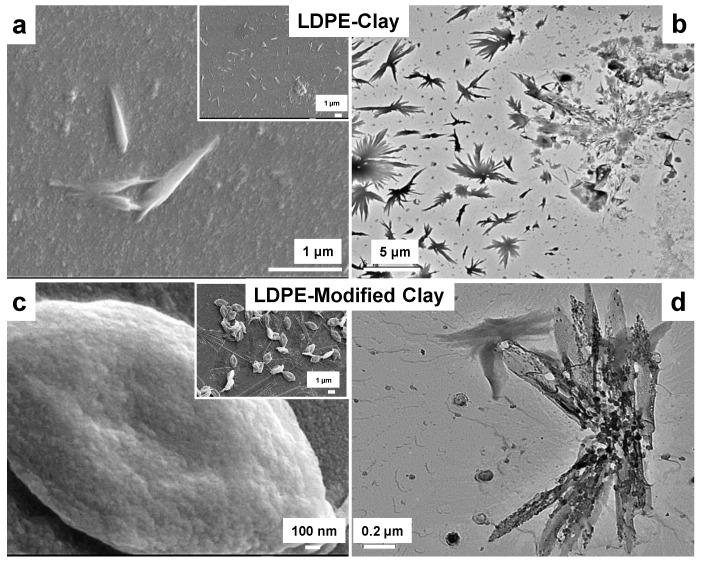
SEM (**a**) and TEM (**b**) micrographs of LDPE-Clay, while (**c**,**d**) are corresponding micrographs of LDPE-modified clay. INSET images are corresponding low magnification images. The magnifications in the micrographs are as: a: 25 k; a’ (INSET): 5 k; b: 7 k; c: 40 k; c’ (INSET): 8 k; and d: 10 k. The SEM chamber pressure varied from 1.4 × 10^−6^ mbar–4 × 10^−6^ mbar.

The morphology of the LDPE-nanofiller composites was observed using a combination of SEM and TEM, and the results are presented Figure 1 and Figure 2, Figure S1. Figure 1a,b represent the SEM and TEM micrographs of LDPE-CNT, respectively. The images show numerous crystals of LDPE nucleated on the long axis of CNTs. It can be observed that the LDPE developed a periodic crystallization pattern in the form of NHSK via epitaxial crystallization [18]. The high magnification TEM image in Figure 1b shows LDPE crystals of ~100 nm in diameter with a thickness of ~30 nm and a distance between adjacent lateral crystals of ~100 nm.

Figure 1c,d shows the SEM and TEM micrographs of LDPE-GO, respectively. The structure of the GO platelets does not produce the NHSK morphology as observed in the LDPE-CNT sample. Instead, numerous petal-shaped LDPE crystals appear to coat the GO nanoplatelets and allows the polymer to continue to grow around the nanoparticle to create anisotropic, globular structures as shown in Figure 1c. This is further confirmed by SEM of pure LDPE crystallized homogeneously (without any nucleating agent), showing a spherulitic growth (Figure 1e). In contrast, LDPE coats GO as globules protruding randomly. Graphene is known to serve as effective nucleating agent at low concentrations, while not having a strong impact on the morphology of the formed crystals [19,20,21,22].

The structural transition of LDPE grown on CNTs in the form of NHSK to a globular, petal-shaped morphology when grown on GO can be explained by considering the effect of the shape of the nucleating surface. The diameter of the nucleating surface is known to be a critical parameter in crystal formation [23]. CNTs have small diameters (2 nm in the present case), so the polymer chains tend to align along the axis inducing nucleation through soft epitaxy, where strict lattice matching is not required [24]. As a result, the LDPE crystal lamellae grow outward in the radial direction perpendicular to the CNT shish with periodic crystals due to numerous nucleation points along the length of the CNT axis. On a surface where the diameter is much larger than the radius of gyration of polymer (~10 nm), geometric confinement is a major factor as the polymer begins to crystallize on the nanofiller where the polymer chains are exclusively parallel to the particle axis [25]. It has been reported previously that, CNTs diameter smaller than 20 nm promoted kebab formation via size-dependent soft-epitaxy, while larger diameter (>150 nm) CNTs followed molecular epitaxy, with polymer crystals growing in multiple orientations [26].

When crystallizing on GO, the platelet represents a flat surface without an axis to which the polymer chains can align. This may allow LDPE to crystallize onto the surface in a parallel fashion through soft epitaxy, which does not produce the same NHSK architecture as seen when nucleating with CNTs. Notably, both CNTs and graphene provided a nucleating surface for heterogeneous crystallization, but in GO, the growth of LDPE occurs with the formation of more globular, space-filling morphologies. GO single sheets are difficult to observe under TEM due to their permeability. However, few stacked layers can be easily seen under TEM. As shown in Figure 1c,d, LDPE primarily coated the graphene sheets.

#### 3.1.2. Polymer Crystal Structure on Clay-Based Nanofillers

Figure 2a,b represent the SEM and TEM micrographs of LDPE-clay, respectively, where a randomly oriented spherulitic growth of LDPE on clay was found. The size of these crystals was in the range of 1–2 µm. On the other hand, large spheroid shaped crystals can be found on LDPE crystallized on modified clay (Figure 2c,d). On a mesoscopic scale, the morphology of LDPE-clay and LDPE-modified clay consists of micron-sized spherulites. This suggests that clay platelets were responsible for the growth of oriented crystals.

The platelets of the unmodified clay remain tightly packed allowing the LDPE to coat and crystallize around a bundle of clay particles. In modified clay, the presence of the modifier allows the LDPE to crystallize around what appear to be single sheets of the nanoparticle, which results in the appearance of the small, thin crystals as seen in Figure 2d. For modified clay, the cationic exchange with quaternary ammonium salt lowers the surface energy of modified clay and the compatibility between modified clay and polymer is improved [27]. This allows more polymer to intercalate inside the parallel clay galleries. Further, the intercalation of polymeric chains inside modified clay is further promoted by slow crystallization at a higher temperature (80 °C) for 30 min. This extended time and high temperature might have assisted in a slower but efficient diffusion of LDPE chains inside modified clay. A larger inter-layer spacing within the modified clay tactoids provides additional room for ordering of lamellas, resulting in larger spherulites.

### 3.2. X-ray Diffraction

**Figure 3:**
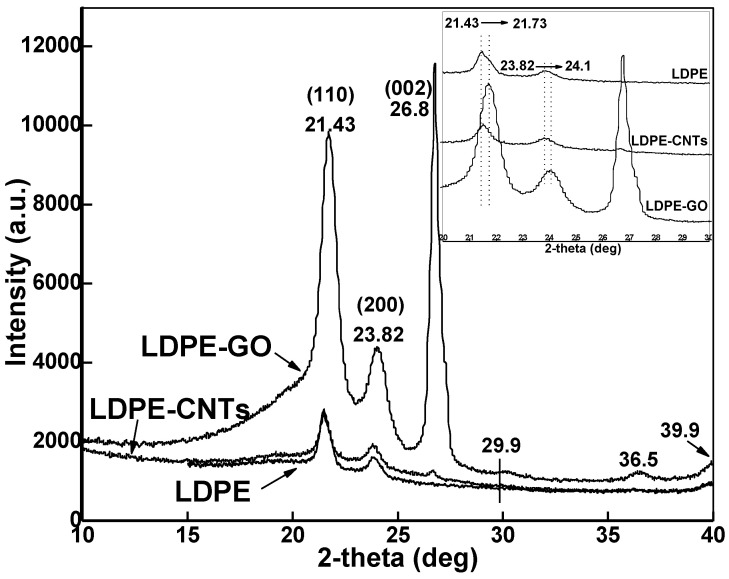
X-ray diffraction patterns of pure LDPE, LDPE-CNTs, and LDPE-GO. INSET image is provided to illustrate peak shift.

The X-ray diffraction (XRD) patterns of pure LDPE and its composites with CNT and GO are given in Figure 3. As seen in the diffraction pattern, two peaks at 2θ = 21.43° (110 reflection) and 23.82° (200 reflection), due to triclinic unit cell of LDPE, were found [28,29]. In the carbon-based fillers (CNTs and GO), there is another sharp peak at 2θ = 26.8° which is believed to be the (002) crystal face of carbon [30]. Now, if we carefully observe the XRD patterns (INSET figure provided for better observation) of LDPE-CNTs and LDPE-GO, it is clear that the peaks were more intense for the LDPE-GO than those of LDPE-CNTs.

A magnified version of XRD patterns (INSET) provides better observation of the peak shift. A slight shift towards a higher 2-theta value in the XRD peak for LDPE was observed from 21.43° to 21.73° in the specimen with GO, which is reflective of penetration of LDPE chains inside nanoplatelets of GO and the resultant exfoliation of the GO layers [31]. On the other hand, no change was observed for LDPE-CNTs, which is indicative of intact tubular structure of CNTs. The peak shift from 21.43° in pure LDPE to 21.73° in LDPE-GO is indicative of conversion of triclinic to orthorhombic form. Further, small peaks at 29.9, 36.5°, and 39.9° are also due to the (210), (020), and (011) diffraction peaks of orthorhombic LDPE, respectively [32].

**Figure 4:**
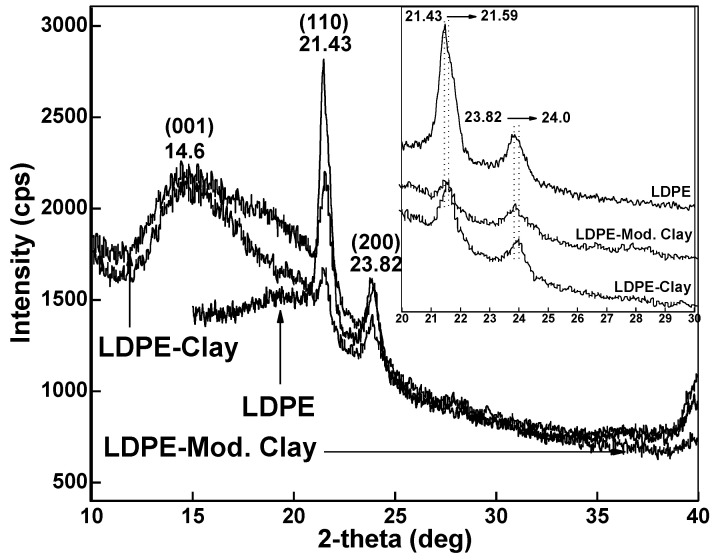
X-ray diffraction patterns of pure LDPE, LDPE-Clay, and LDPE-modified clay. INSET image is provided to illustrate peak shift.

The XRD patterns of LDPE-clay nanocomposites are shown in Figure 4 with two sharp peaks at 21.43° and 23.82° from LDPE. A broad peak at 2θ = 14.6° was found, which is attributed to clay and modified clay. X-ray scans of LDPE-clay and LDPE-modified clay nanocomposites have peaks similar to the pure LDPE but shifted to a higher 2θ representing a larger d-spacing. The peak shift indicates that the nanofiller gallery (space between layers) has expanded and it is generally assumed that the amorphous polymer matrix has intercalated the nanostructure [33,34].

The crystallinity was calculated by dividing the total area of the LDPE crystalline peaks by the total area under the diffraction curve. Pure LDPE had a crystallinity of 59.7%. The percent crystallinity of the LDPE-CNT was 34.4%, while LDPE-GO had a crystallinity of 56.7%. LDPE-Clay and LDPE-modified clay had % crystallinities of 48.23% and 57.1%, respectively. Notably, in all the cases, the % crystallinity was decreased as compared to the crystallinity of pure LDPE, which is due the nucleating agents inhibiting ordering of polymer chains through geometric confinement, hence decreasing the overall crystallinity. The increase in crystallinity when using modified clay instead of natural clay may be attributed to the increase in d-spacing between clay layers, which allows more polymer into the modified clay gallery, as well as the higher specific surface area which can nucleate more crystals [35,36]. Crystallinity followed the trend as: LDPE-CNTs < LDPE-Clay < LDPE-GO < LDPE-Modified Clay < Pure LDPE.

### 3.3. Chemical Structure Analysis of Crystallized Polymer by FTIR

The chemical structure and crystal morphology of polymers nucleated in the presence of nanoparticles is known to depend on their interaction [37,38]. Fourier transform infrared spectroscopy is an effective technique to study these nanoparticle–polymer interactions.

#### 3.3.1. Carbon-Based Nanofillers

**Figure 5:**
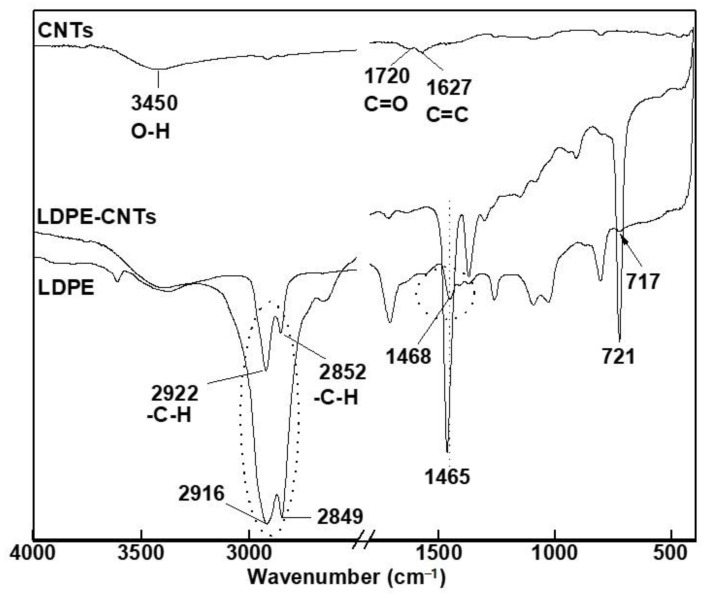
FTIR spectrum of pure CNTs, pure LDPE, and LDPE-CNTs.

The characteristic FTIR spectra of CNTs, LDPE, and LDPE-CNTs are presented in Figure 5. In the FTIR spectra of CNTs, the peaks at 1720 and 1627 cm^−1^ represent -C=O and -C=C- stretching vibrations, respectively, primarily due to the acid treatment during purification. The presence of a small and broad peak at around 3450 cm^−1^ represents O-H stretching vibrations from absorbed moisture.

In the spectra of pure LDPE, the two split peaks at 2916 and 2849 cm^−1^ are assigned to the asymmetric and symmetric stretching vibration bands of -CH_2_-; the peaks at 1465 and 721 cm^−1^ correspond to the deformation vibration band and in-plane rocking vibration band of -CH_2_-, respectively [39]. In contrast, the direct crystallization of LDPE on the long axis of CNTs (LDPE-CNTs) indicates a small shift of the asymmetric and symmetric stretching vibration band (-CH_2_-) from 2916 and 2849 cm^−1^ in PE to 2922 and 2852 cm^−1^, respectively, in the CNT-LDPE nanohybrid structure. This shift is ascribed to the favorable physical interaction between PE and CNTs [40]. Furthermore, it also suggests noncovalent and nonspecific -CH-π interactions between PE and CNTs lead to crystallization along the axis of the CNTs.

It has been suggested that the physical interaction of the -C-H group of a polymer with CNTs is expected to result in broadening, shift and/or split in the frequency of the -C-H stretching and bending vibrations of the polymer [41]. As a consequence of non-covalent or non-specific -CH-π interactions, the polymer may wrap around the CNTs.

**Figure 6:**
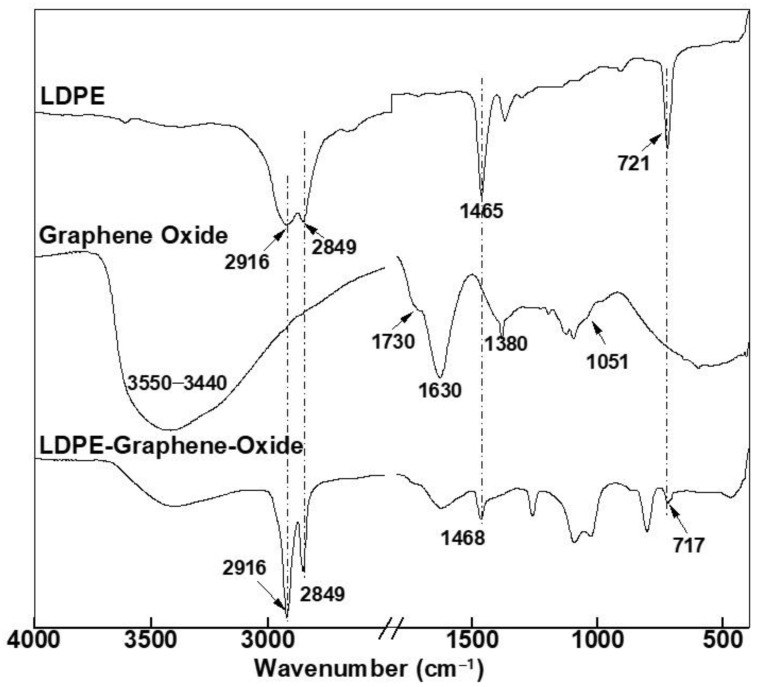
FTIR spectrum of pure GO, pure LDPE, and LDPE-GO.

The FTIR spectra of GO (Figure 6) is characterized by the presence of the oxygen-containing functional groups. The peaks at 1051, 1380, and 1630 cm^−1^ correspond to the -C-O-C- stretching vibration, -C-OH stretching, and -C=O stretching modes of the quinone skeleton of graphene, respectively, while peaks at 1730 and 3455 cm^−1^ correspond to -C=O stretching vibrations of the -COOH groups and -O-H stretching vibrations, respectively.

If we now consider the FTIR spectra of LDPE-GO and LDPE-CNTs, and compare with pure LDPE, we observe that in the spectra of LDPE-GO (Figure 6), the peaks due to symmetric and asymmetric vibrations (2916 and 2849) remained unchanged in terms of peak shift. However, these peaks become more distinct, which indicates an increase in crystallinity [42]. Further, the peak at 1465 in LDPE has been shifted to 1468, while the peak at 721 has been shifted to 717, indicating chemical interactions between -CH_2_- group of PE and GO. A previous study indicated absence of any chemical interactions between PE and graphene [43]. However, in our case, GO, owing to the functional groups, possesses stronger and more complex chemical interactions between LDPE and GO, resulting in an increase in crystallinity and peak shifts.

#### 3.3.2. Clay-Based Nanofillers

**Figure 7:**
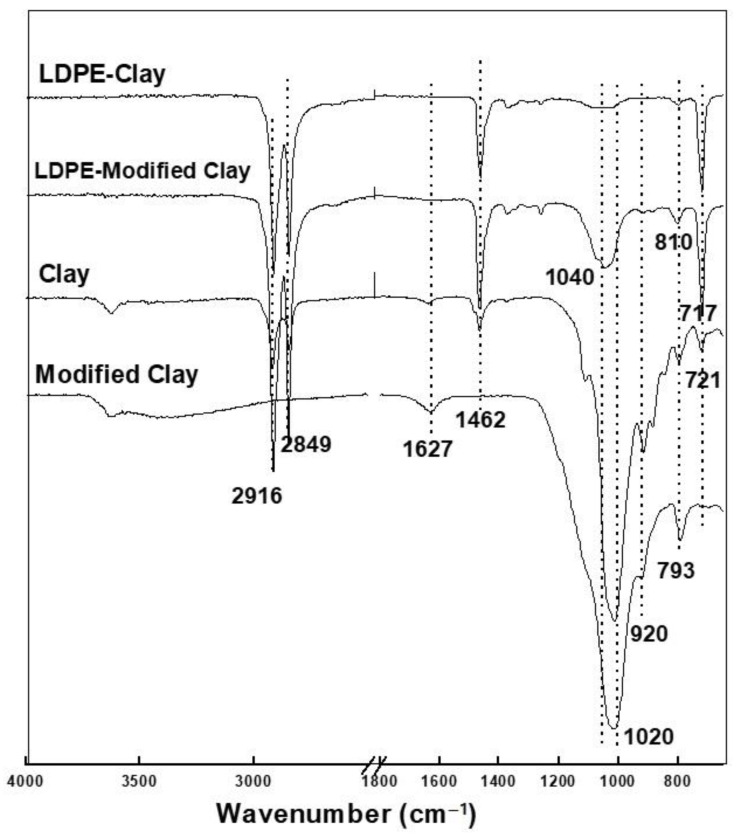
FTIR spectrum of pure clay, pure modified clay, LDPE-Clay, and LDPE-modified clay.

The FTIR spectra of pure clay, modified clay, and their LDPE nanohybrids are given in Figure 7. The peak at ~3650 cm^−1^ is assigned to the hydroxyl (-OH) group of moisture in the clay, while the peaks at 1627 and 1020 cm^−1^ are due to -OH deformation of water (H-O-H bending mode) and Si-O stretching vibrations in clay.

The peaks at 920 and 793 cm^−1^ correspond to Al-Al-OH deformation and Si-O stretching vibrations [44]. No significant difference was found on the FTIR spectra of natural clay and modified clay, except for the peaks at 2916 and 2849 cm^−1^ assigned to the amine group (-NH_2_) stretching vibrations in modified clay. The FTIR spectra of LDPE-clay and LDPE-modified clay were significantly different than the FTIR spectra of pure LDPE, while the FTIR spectra of LDPE-clay and LDPE-modified clay were very similar. The peak at 1465 cm^−1^ in LDPE has been shifted to 1462 cm^−1^ in its clay composite. These observations can be attributed to the ion-dipole interactions of the interlayer cation of clay and the -CH_2_- (methylene) units in LDPE.

The methylene (-CH_2_-) group of PE is very sensitive to conformational change, which in turn is related to the chemical interactions between the methylene group and the nanofillers. PE crystals are primarily found in a monoclinic-like phase. The orthorhombic phase of PE is more stable thermodynamically, but the monoclinic phase has previously been reported as a result of plastic deformation of orthorhombic-like phase [45]. In the absence of any ordered chain arrangements, the methylene rocking band appears at 721 cm^−1^, as also indicated in the FTIR spectra of pure LDPE. However, the presence of a nucleating agent or a nanofiller can promote short range ordering, which can split this band in such a way that monoclinic arrangement appears at 717 cm^−1^ and orthorhombic appears at 731–719 cm^−1^ [46]. Similarly, the methylene bending modes appear at 1463–1461 cm^−1^ (short trans amorphous sequences) and 1468–1465 cm^−1^ (long trans disordered/amorphous sequences) [47].

**Figure 8:**
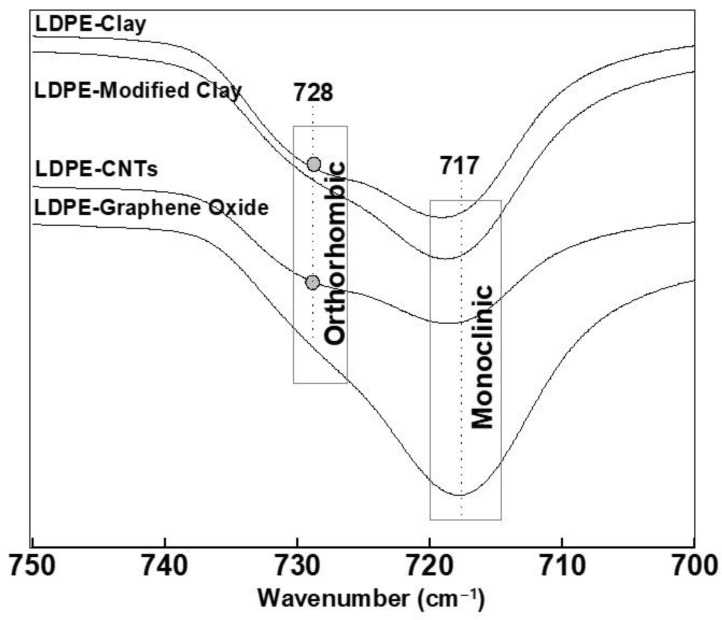
FTIR spectrum of LDPE-based nanohybrids in spectral region of 750–700 cm^−1^.

Figure 8 shows the FTIR spectra of methylene rocking vibrations (from 750–700 cm^−1^) of LDPE crystallized with various nanofillers. An intense absorption at 717 cm^−1^ was found for all of the samples. This indicates that PE in all the cases is predominantly monoclinic. However, a small absorption band at 728 cm^−1^ was found in LDPE-CNTs and LDPE-clay, which is due to small fraction of orthorhombic content. The peak shift also suggests a decrease in crystallinity in LDPE-CNTs and LDPE-clay, which is in agreement with the crystallinity data obtained using XRD.

These results suggest an interaction between LDPE and the nanofillers. PE can grow epitaxially on GO and modified clay and can also exfoliate these nanofillers to allow more polymer impregnation [48]. These results clearly indicate that the epitaxially grown PE on GO contains more monoclinic crystal structure than LDPE on CNTs. On the other hand, natural clay, being tightly stacked with limited d-spacing, restricts the motion of the PE chains. Hence, during crystallization, the PE chains experience reduced viscous flow while intercalated between the lamellar clay tactoids and reduced volume for growth. Under these conditions, the crystallization will proceed very slowly due to the lower mobility of polymer chains inside pure clay tactoids [49]. This results in a small fraction of orthorhombic polymer crystal structures in LDPE-clay. On the other hand, modified clay can provide a more open space for relaxation to produce a monoclinic conformation by providing enough space inside the modified clay galleries.

**Figure 9:**
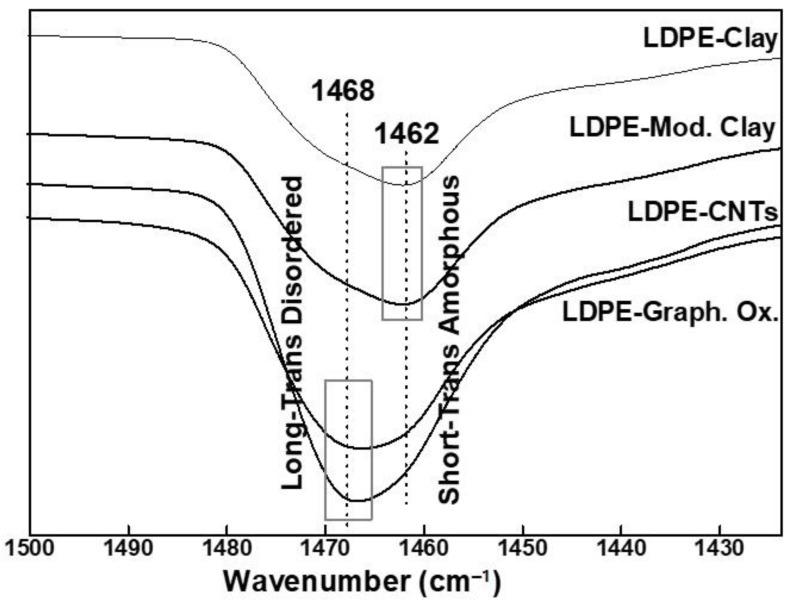
FTIR spectrum of LDPE-based nanohybrids in spectral region of 1500–1420 cm^−1^.

Figure 9 shows the FTIR spectra of LDPE nanohybrids in the methylene bending vibrational region (1500–1420 cm^−1^). The spectra of LDPE-CNTs and LDPE-GO were similar with a small peak at 1462 cm^−1^ due to the amorphous phase. The spectra of LDPE-clay and LDPE-modified clay were similar with a peak around 1468 cm^−1^, which is ascribed to the crystalline orthorhombic phase. A similar result was recently reported in terms of orthorhombic phase of PE on graphene [50]. PE chains align on tubular CNTs in such a way that their preferential interactions lead to a stronger constraint on chain relaxation, giving rise to orthorhombic phase [51]. This is in close agreement with other reports that explained the orientation of amorphous PE on CNTs, in the absence of any extensional flow [52]. Similarly, in the case of clay, the tightly stacked platelets allow the PE chains to intercalate, but due to restricted inter-lamellar space, the movement of PE chains through interstices is limited. This constrained molecular motion in a confined volume gives rise to more amorphous regions, which was detected in the FTIR spectra at 1462 cm^−1^.

## 4. Conclusions

The solution crystallization of LDPE on CNTs, GO nano-platelets, clay, and modified clay was investigated through morphological and FTIR analysis. GO promoted epitaxial growth and crystalline monoclinic conformation, while tubular CNTs hinders the crystal orientation and gives rise to an amorphous orthorhombic phase. Similarly, tightly stacked natural clay restricted free movement of PE chains and limited the area available for nucleation, giving rise to more amorphous phase, while modified clay will have crystalline monoclinic PE due to the mobility of the polymer chains inside their layers. XRD analysis revealed that the modified clay samples also produced very high levels of crystallinity even though LDPE is difficult to crystallize. Noncovalent and non-specific –CH-π interactions between PE and the nucleating agents determine the conformation of crystallized polymer. Our results indicated that the shape of the nucleating agent is more crucial than its specific surface area, in crystallizing a polymer. PE crystallized differently on various nanofillers and exhibited different crystalline polymorphs, crystallinity, and microstructures. This fundamental understanding of how various nanofillers dictate the polymer microstructure can be used to control the nucleation and growth of polymer crystal in the fabrication of high-performance polymer nanocomposites so as to tune the resultant properties.

## Data Availability

The data presented in this study are available on request from the corresponding author.

## Supplementary Materials

The following are available online at https://www.mdpi.com/article/10.3390/polym13101558/s1, Figure S1: Atomic force micrograph (a) and height profile (b) of pure graphene oxide.
