# Supplementary figures and images for: Morphological and Chemical Analysis of Low-Density Polyethylene Crystallized on Carbon and Clay Nanofillers
Source: Polymers (Basel). 2021 May 13;13(10):1558. doi: 10.3390/polym13101558 (PMC8152291; doi:10.3390/polym13101558)

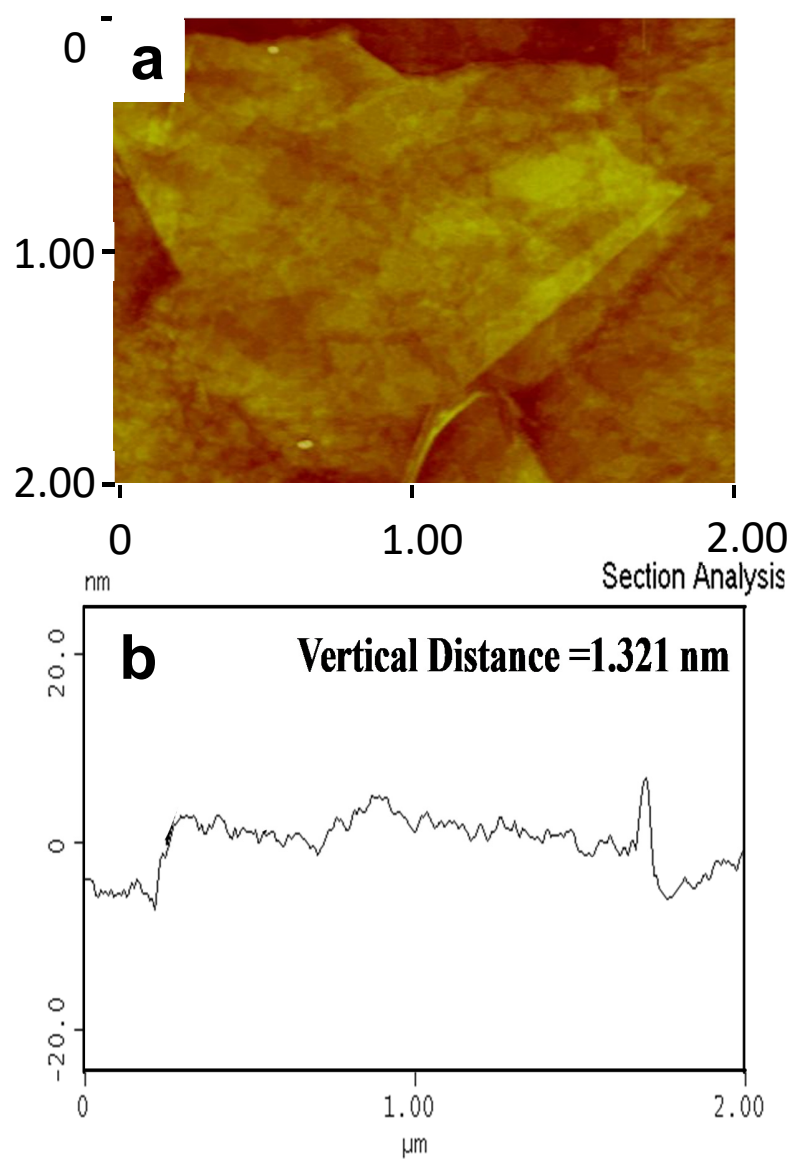

**Figure S1.** Atomic force micrograph (a) and height profile (b) of pure graphene oxide.

Supplement: Supplementary file 1 [file polymers-13-01558-s001.zip › polymers-1203401-SI.pdf]
